# Knockdown of Golgi phosphoprotein 2 inhibits hepatocellular carcinoma cell proliferation and motility

**DOI:** 10.18632/oncotarget.7271

**Published:** 2016-02-08

**Authors:** Yiming Liu, Xiaodi Zhang, Ting Sun, Junchang Jiang, Ying Li, Mingliang Chen, Zhen Wei, Weiqin Jiang, Linfu Zhou

**Affiliations:** ^1^ Medical Biotechnology Laboratory, Zhejiang University School of Medicine, Hangzhou 310058, China; ^2^ Department of Pathology, Zhejiang Provincial People's Hospital, Hangzhou 310014, China; ^3^ Department of Pathology, RunRun-Shaw Hospital Affiliated to Zhejiang University School of Medicine, Hangzhou 310016, China; ^4^ Clinical Laboratory, Children's Hospital Affiliated to Zhejiang University School of Medicine, Hangzhou 310003, China; ^5^ Laboratory Animal Center, Zhejiang University School of Medicine, Hangzhou 310058, China; ^6^ Cancer Center, First Affiliated Hospital, Zhejiang University School of Medicine, Hangzhou 310003, China

**Keywords:** Golgi phosphoprotein 2, hepatocellular carcinoma, siRNA, cell proliferation, biomarker

## Abstract

Golgi phosphoprotein 2 (GP73) is highly expressed in hepatocellular carcinoma (HCC) cells, where it serves as a biomarker and indicator of disease progression. We used MTS assays, anchorage-independent cell colony formation assays and a xenograft tumor model to show that GP73-specific siRNAs inhibit HCC proliferation in HepG2, SMMC-7721, and Huh7 cell lines and *in vivo*. Following GP73 silencing, levels of p-Rb, a factor related to metastasis, were reduced, but cell cycle progression was unaffected. Our results suggest that GP73 silencing may not directly suppress proliferation, but may instead inhibit cell motility. Results from proliferation assays suggest GP73 reduces expression of epithelial mesenchymal transition (EMT)-related factors and promotes cell motility, while transwell migration and invasion assays indicated a possible role in metastasis. Immunofluorescence co-localization microscopy and immunoblotting showed that GP73 decreases expression of N-cadherin and E-cadherin, two key factors in EMT, which may in turn decrease intracellular adhesive forces and promote cell motility. This study confirmed that GP73 expression leads to increased expression of EMT-related proteins and that GP73 silencing reduces HCC cell migration *in vitro*. These findings suggest that GP73 silencing through siRNA delivery may provide a novel low-toxicity therapy for the inhibition of tumor proliferation and metastasis.

## INTRODUCTION

Golgi phosphoprotein 2 (GP73) is a highly-phosphorylated protein located in the *cis* and medial-Golgi apparatus [1]. Under steady-state conditions, GP73 is an integral membrane protein expressed at only low levels. However, it is highly expressed in hepatic, colon, cervical and gastric carcinomas, with higher levels correlated with disease progression [2]. The N-terminal region of GP73 contains a transmembrane domain (TMD) that can be cleaved by proprotein convertase, resulting in the release of GP73 into the extracellular space [3, 4]. This secretory GP73 can be detected in serum, and in recent years has been utilized as a novel biomarker in clinical diagnoses [2–4]. When compared with the more conventionally-utilized biomarker, alpha-fetoprotein (AFP), GP73 provides enhanced detection sensitivity. Moreover, serum GP73 concentrations increase during early-stage cancer, while AFP concentrations remain essentially unaltered [5–7]. Additionally, GP73 is upregulated both during and after adenoviral, HBV, HCV, or HIV infection, thus making it a potential viral biomarker [1, 8–11].

While an increasing number of studies have associated GP73 with cancer diagnosis, little has been reported with respect to liver diseases, immune system functions or tumor proliferation and metastasis [3, 12]. In the present study, the potential role of GP73 in hepatocellular carcinoma (HCC) cell proliferation and motility was examined using silencing.

## RESULTS

### siGP73 downregulated GP73 expression *in vitro*

**Figure 1:**
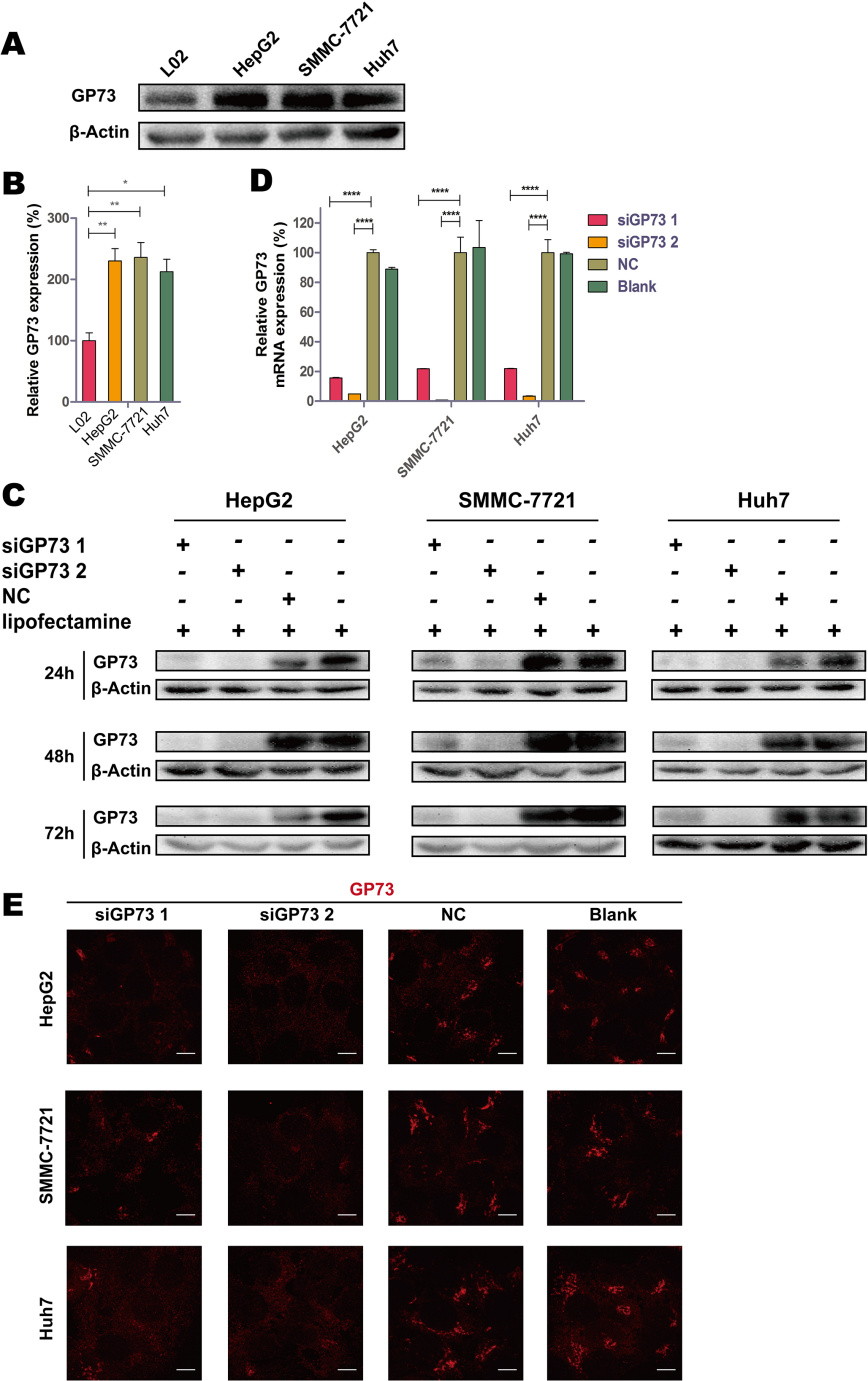
GP73 is highly expressed in HCC cells, and siGP73 downregulated GP73 expression *in vitro* Expression of GP73 in L02, HepG2, SMMC-7721 and Huh7 cells analyzed by Western bolt (**A**). Expression of L02, HepG2, SMMC-7721 and Huh7 cells were analyzed by Mann-Whitney *U* test (**B**). GP73 expression in cells treated with GP73 siRNA (300 nM) for 24, 48 and 72 h (**C**). GP73 mRNA expression analyzed by RT-qPCR at 72 h (**D**). GP73 was visualized by immunostaining and confocal microscopy (**E**). Scale size: 10 μm. Data were normalized to the highest expression group, and were shown as means ± SEM (*n* = 3). *p < 0.05, **p < 0.01, ****p < 0.0001.

Compared with the normal liver cell line L02, GP73 was highly expressed in the HCC cell lines HepG2, SMMC-7721 and Huh7 (Figure 1A). GP73 silencing efficiency was analyzed by immunoblotting at 24, 48 and 72 h (Figure 1C). RT-qPCR results showed that silencing was greatest at 72 h, with 95.06% ± 0.03%, 99.35% ± 0.03% and 96.56% ± 0.18% knockdown in the HepG2, SMMC-7721, and Huh7 cell lines, respectively (Figure 1D). Following silencing, GP73 was also visualized in cells via immunohistochemical staining (Figure 1E).

### GP73 silencing downregulated p-Rb expression, with little effect on cell cycle progression

**Figure 2:**
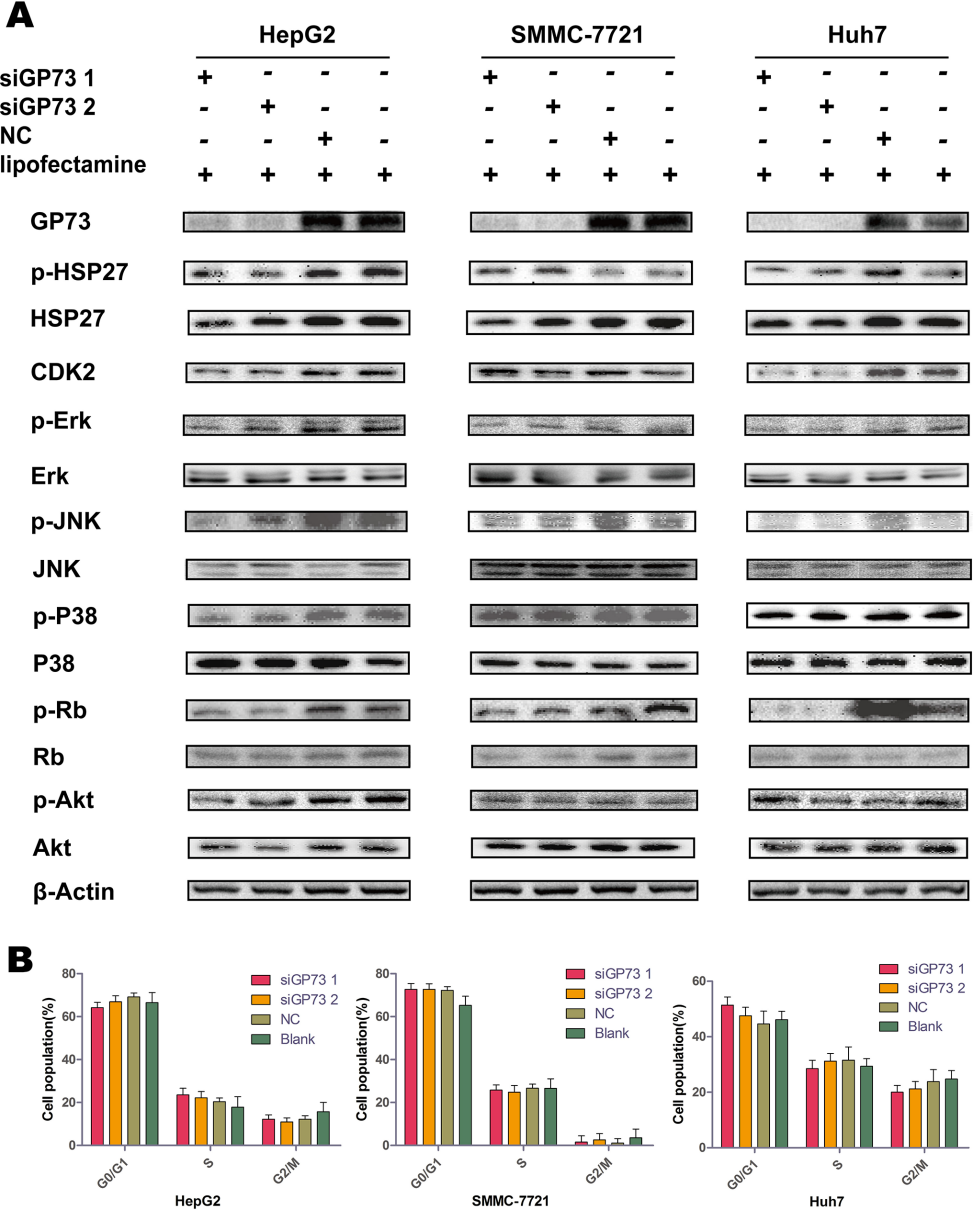
GP73 silencing downregulated p-Rb but did not impact cell cycle progression Expression of MAPK and PI3K/Akt pathway factors and cell cycle-related proteins 72 h post-siRNA treatment (**A**). Cell cycle progression was analyzed by flow cytometry after siRNA treatment (**B**).

Following GP73 knockdown, Mitogen-activated protein kinase (MAPK) pathway and cell cycle-related factors were examined via immunoblotting (Figure 2A). p-Rb was sharply downregulated in all cell lines. HSP27 and CDK2 were downregulated in the HepG2 and Huh7 lines, but were upregulated in SMMC-7721 cells, possibly as a result of cellular heterogenicity in the SMMC-7721 cell line.

Due to the role of p-Rb in cell cycle regulation, we hypothesized that the cellular distributions of each phase of the cell cycle would change as a result of GP73 silencing. However, flow cytometry showed that there were no significant changes in cell cycle progression between the silenced and control groups in all three cell lines (Figure 2B).

### GP73 silencing inhibited cell proliferation *in vitro*

**Figure 3:**
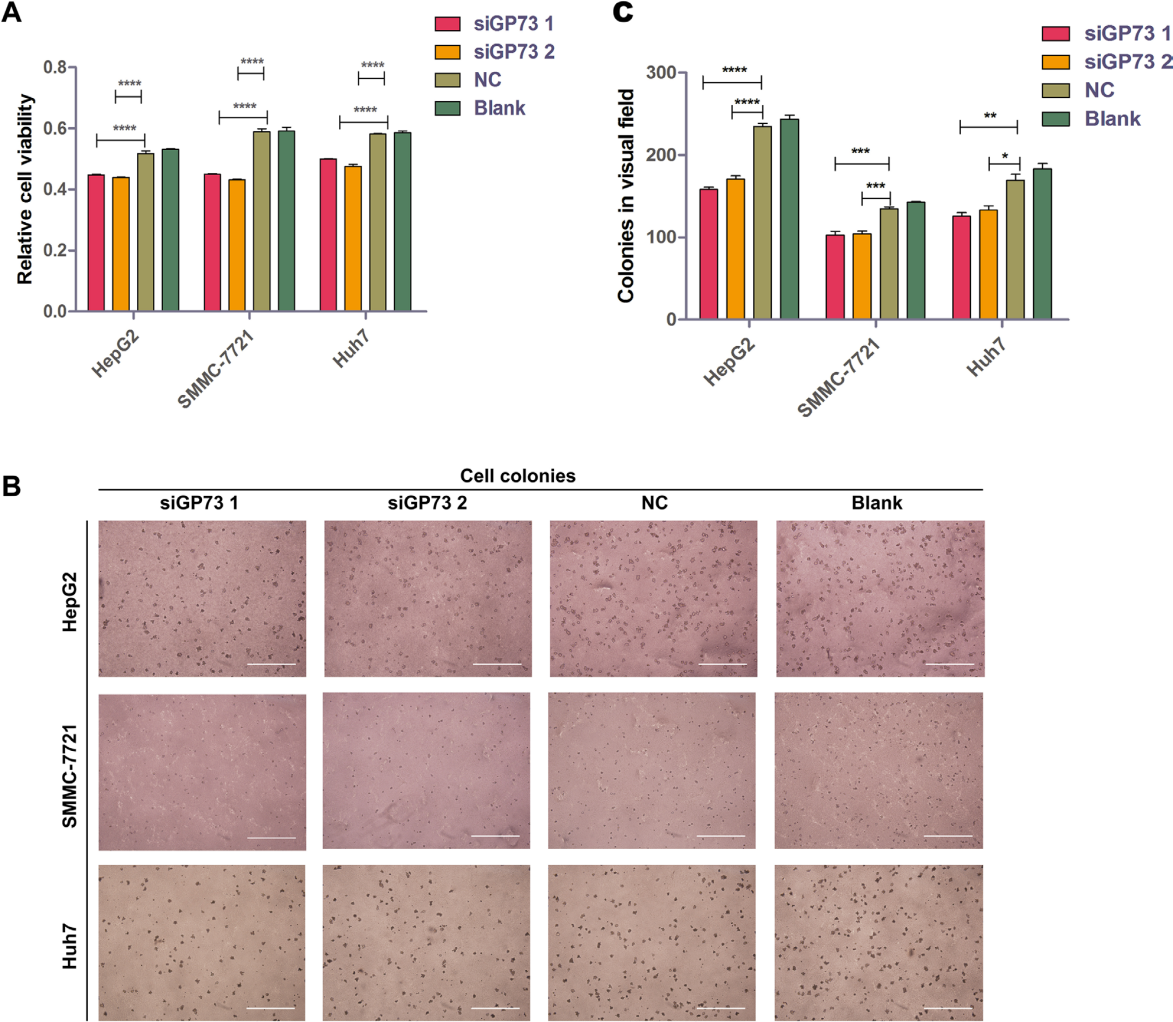
GP73 silencing inhibited cell proliferation *in vitro* Cell viability as determined by OD measurement (**A**). and colony formation (diameter > 100 μm in HepG2 and Huh7 cells or > 50 μm in SMMC-7721 cells) (**B** and **C**). in HepG2, SMMC-7721 and Huh7 cells following siRNA treatment. Colonies were counted after six days. Scale size: 1000 μm. Data are shown as means ± SEM (*n* = 3). *p < 0.05, **p < 0.01, ***p < 0.001, ****p < 0.0001.

To investigate whether GP73 silencing in HepG2, SMMC-7721 and Huh7 cell lines could inhibit cell proliferation, cell viability was quantified via MTS assay following siGP73 transfection. We found that siGP73 treatment reduced viability in HepG2 (17.30% ± 0.21%), SMMC-7721 (27.07% ± 1.14%) and Huh7 (18.79% ± 1.93%) cell lines (Figure 3A). In an anchorage-independent cell colony formation assay, GP73 inhibition reduced colony formation in HepG2, SMMC-7721 and Huh7 cells by 35.16% ± 3.35%, 28.04% ± 7.93% and 31.33% ± 6.08%, respectively (Figure 3C).

### GP73 silencing inhibited cell proliferation *in vivo*

To assess whether GP73 silencing could suppress proliferation *in vivo*, we established a human hepatocellular xenograft tumor model in nude mice. We silenced GP73 by modified siRNA *in vivo* instead of using stably-transfected cells.

**Figure 4:**
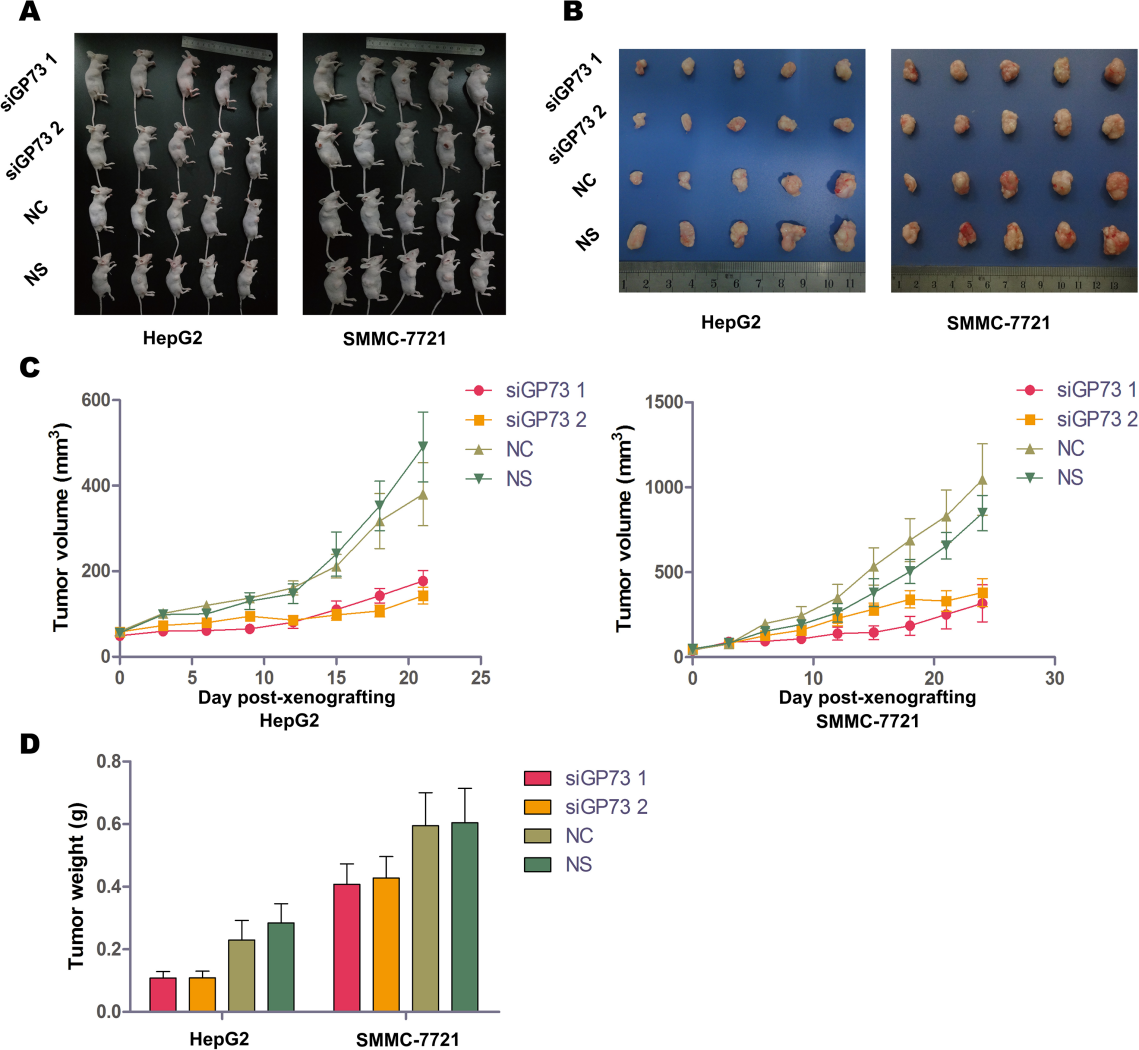
GP73 silencing inhibited cell proliferation *in vivo* HepG2 (left) and SMMC-7721 (right) xenograft-bearing mice treated with siRNA showed no significant toxicity (**A**). HepG2 (left) and SMMC-7721 (right) xenograft tumors at the final siRNA treatment (**B**). Mice were treated with cholesterol-modified GP73 siRNA or NC siRNA at a dose of 1 mg/kg every three days. Growth curves of HepG2 (left) and SMMC-7721 (right) xenograft tumors after treatment with cholesterol-modified GP73 siRNA (**C**). HepG2 (left) and SMMC-7721 (right) xenograft tumor weights at the final treatment time point. Data are shown as means ± SEM (*n* = 5).

**Figure 5:**
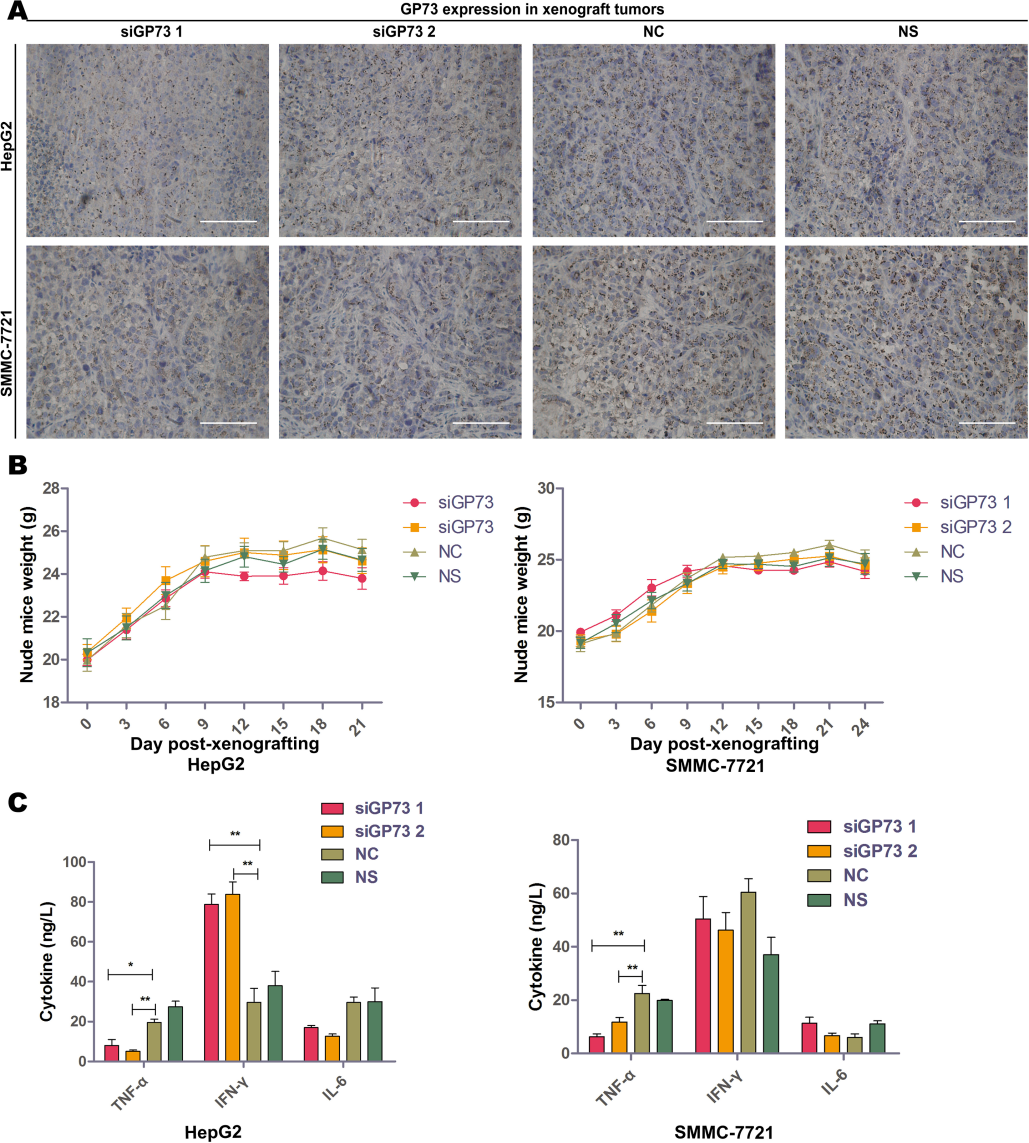
Modified siRNA effectively silenced GP73 *in vivo* with no serious immunoreactions Silencing efficiency of cholesterol-modified GP73 siRNA on HepG2 (upper) and SMMC-7721 (lower) xenograft tumors were measured by immunohistochemistry staining (**A**). Scale size: 100 μm. Body weight changes in HepG2 (left) and SMMC-7721 (right) xenograft-bearing mice (**B**). TNF-α, IFN-γ and IL-6 levels in serum of HepG2 (left) and SMMC-7721 (right) xenograft-bearing mice as measured by ELISA, following the last siRNA treatment (**C**). Data are shown as means ± SEM (*n* = 5), *p < 0.05, **p < 0.01.

HepG2 and SMMC-7721 xenograft tumor volumes and weights were determined two days after the final treatment (Figure 4B–4D). Immunohistochemical analysis demonstrated significant GP73 silencing compared to negative controls (NC) (Figure 5A). Tumor volumes were reduced in HepG2 (57.82% ± 7.82%) as compared to SMMC-7721 xenografts (30.36% ± 12.67%). Approximately 12 days post-treatment, the weights of all mice had decreased slightly (Figure 5B), with no significant differences between the NC and GP73 silencing groups. Otherwise, no visible side effects were noted following GP73 silencing. Analysis of plasma cytokines in HepG2 xenografts showed that TNF-α (71.90% ± 19.41%) and IL-6 (50.34% ± 18.80%) were downregulated, while IFN-γ (140.74% ± 15.91%) was upregulated (Figure 5C). However, while TNF-α (57.61% ± 14.36%) was downregulated in SMMC-7721 xenografts, IL-6 and IFN-γ were unaltered.

### GP73 silencing inhibited cell migration and invasion *in vitro* and upregulated N-cadherin and E-cadherin

**Figure 6:**
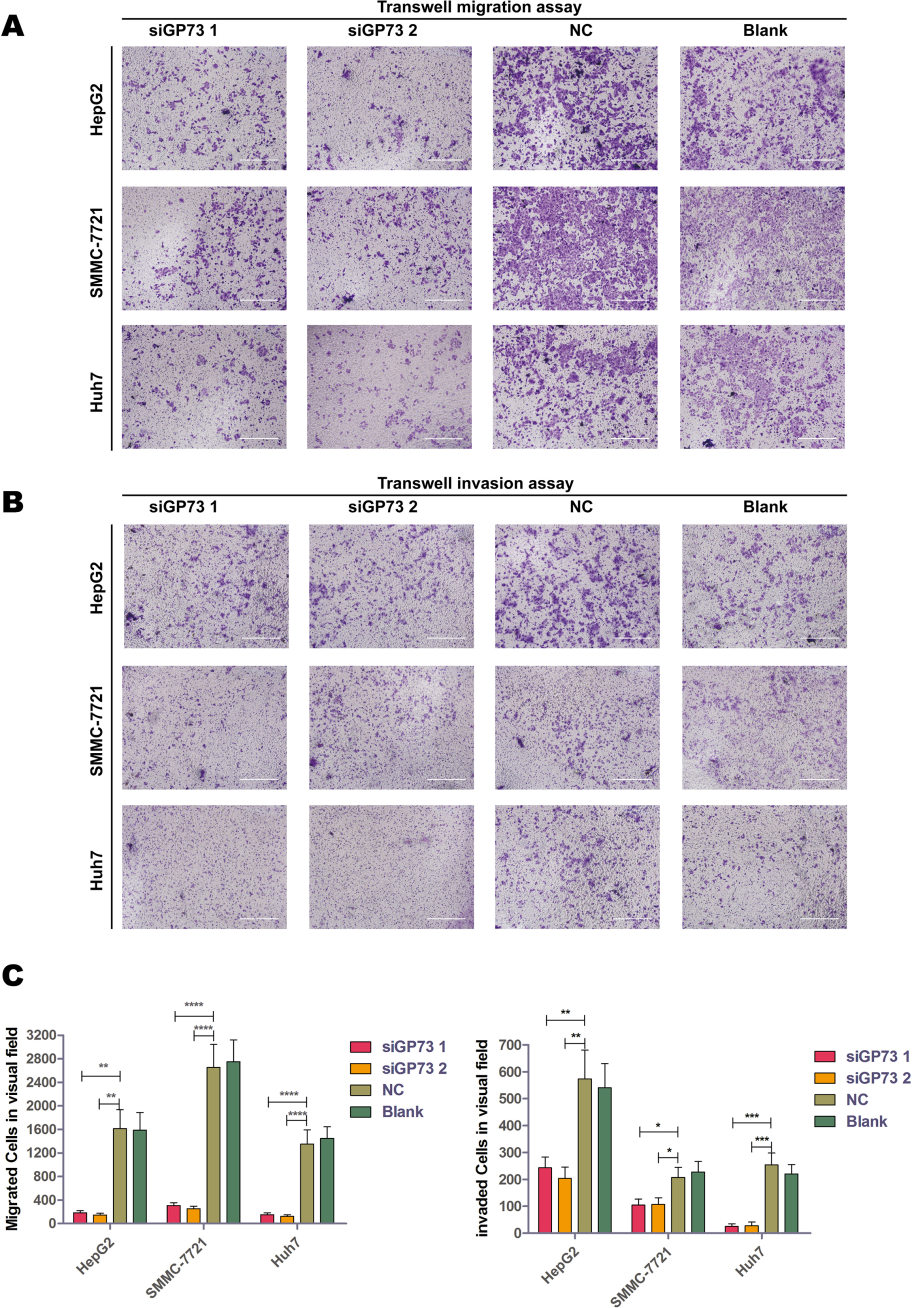
GP73 silencing inhibited cell migration *in vitro* Transwell migration (**A**). and invasion (**B**). assays with HepG2, SMMC-7721 and Huh7 cells. Scale size: 10 μm. Migrated and invaded cells were analyzed by Mann-Whitney *U* test (**C**). *p < 0.05, **p < 0.01, ***p < 0.001,****p < 0.0001.

Transwell migration and invasion assays showed that GP73 silencing significantly suppressed cell migration and invasion in all three cell lines tested (Figure 6A–6B). Migration appeared to be inhibited more than invasion, which suggests that GP73 silencing upregulates EMT-related factors rather than downregulating invasion-related factors.

**Figure 7:**
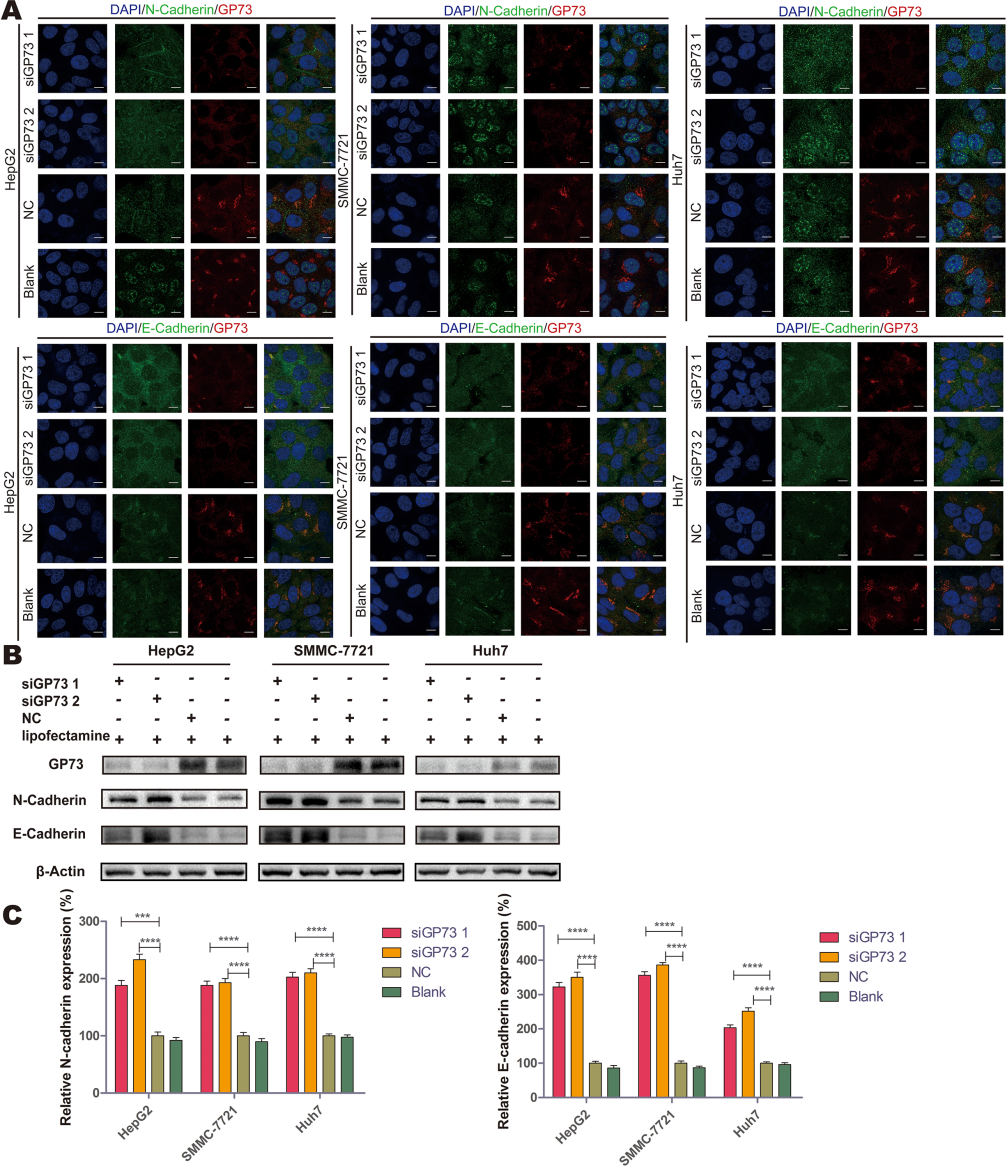
GP73 silencing upregulated N-cadherin and E-cadherin Expression of GP73 (red) and N-cadherin (green) or E-cadherin (green) was visualized by confocal microscopy (**A**). Scale size: 10 μm. N-cadherin and E-cadherin expression at 72 h post-treatment (**B**). Grey scales were analyzed by Mann-Whitney *U* test (**C**). ***p < 0.001,****p < 0.0001.

Immunofluorescence and immunoblotting analyses showed that N-cadherin and E-cadherin, which are important in cell adhesion and the prevention of metastasis, localized in the plasma membrane; the expression of these cadherins increased after GP73 silencing in all three cell lines (Figure 7A and 7B). These results indicate that GP73 may accelerate the process of cell migration by decreasing N-cadherin and E-cadherin expression and promoting tumor proliferation indirectly.

## DISCUSSION

Metastasis is commonly associated with hepatic carcinomas, colorectal carcinomas, cervical carcinomas, lung carcinomas and gliomas, especially in terminal stages. GP73 is highly expressed in these carcinomas, suggesting that GP73 enhances cell migration. This study is the first to reveal that GP73 expression accelerates the process of EMT.

In agreement with other studies, MTS assay showed that GP73 silencing inhibited tumor proliferation [12]. However, we found that the ratio of suppression was much lower than previously reported. Therefore, the present study utilized a soft agar assay as an additional approach to measuring tumor proliferation and anchorage-independent growth. Colony formation inhibition efficiency was somewhat higher than in the MTS assay. Soft agar assay is a method to mimic cell culture *in vivo*; to verify these results, xenografts were generated in nude mice.

Initially, HepG2 cells were transfected with shGP73 prior to injection into mice. However, different cell colonies demonstrated different proliferation rates, resulting in error rates that could not be mitigated even with negative controls. Nude mice could also have been directly injected with PEG-PLA-covered siRNA as previously described, but the nanoparticles were difficult to synthesize [14, 15]. Instead, chemically modified siRNAs were utilized as a more effective alternative [16]. To enable passage through the plasma membrane and to minimize degradation, siRNAs were modified with a 5′-cholesterol and 2′-OMe. These modifications also aided in minimizing the expression of interferons.

As expected, a higher suppression ratio was seen in the HepG2 xenografts compared with the results in soft agar assay following GP73 silencing. The suppression ratio was relatively lower in SMMC-7721 xenografts than in HepG2 xenografts, which might have been the result of rapid tumor generation and insufficient siRNA dose.

To further characterize the effects of GP73 on tumor proliferation, key proteins related to cell proliferation, carcinogenesis and cell cycle progression were examined, including proteins in the MAPK and PI3K/Akt signaling pathways. However, only p-Rb was significantly downregulated as a result of GP73 silencing. Moreover, cell cycle progression was unaffected, suggesting that GP73 might not affect proliferation directly.

Proliferation assays showed that the proliferation suppression ratio increased *in vivo*, possibly as a result of EMT inhibition. In agreement with a previous study, our results demonstrated that p-Rb downregulation could inhibit cell migration [17]. Therefore, we hypothesized that GP73 could accelerate the process of cell migration. In transwell migration and invasion assays, migration was significantly reduced by GP73 knockdown as compared to negative controls. Invasion was also reduced but not to the same extent.

To further examine the association of GP73 knockdown and cell migration, the EMT-related proteins N-cadherin and E-cadherin were examined. Cadherins are associated with cell junctions and are expressed at only low levels in most cancer tissues [13], thus weakening cell adhesions and promoting migration. The results of GP73/cadherin immunofluorescence co-localization microscopy and immunoblot assays showed that GP73 knockdown increased expression of these cadherins.

The present study showed that delivery of cholesterol-modified siRNA results in a high silencing efficiency *in vivo*, as shown via immunohistochemistry. Weight analyses and the appearances of nude mice indicated little-to-no cell cytotoxicity as a result of treatment. Overall, these findings suggest that GP73 silencing may serve as a potential therapy for the treatment of malignant tumors.

## MATERIALS AND METHODS

### Cell culture

Human hepatocellular carcinoma cell lines HepG2, SMMC-7721, Huh7 and human normal liver cell line L02 were purchased from American Type Culture Collection (ATCC). Cells were maintained in Dulbecco's Modified Eagle medium (DMEM) high glucose medium (Hyclone, Logan, USA) with 5% fetal bovine serum (FBS) (Biological Industries, Kibbutz Beit Haemek, Israel) and grown in complete medium (DMEM high glucose medium with 10% FBS) at 37°C and 5% CO_2_.

### siRNA

All siRNA duplexes targeting GP73 and negative control siRNAs were purchased from GenePharma Co. (Shanghai, China), with sequences as follows:

siGP73 1: sense, 5′-GUGGCUUAGAAUUUGAA CATT-3′ and antisense, 5′-UGUUCAAAUUCUAAGCC ACTT-3′; siGP73 2: sense, 5′-CAAGCUGUACCAG GACGAATT-3′ and antisense, 5′-UUCGUCCUGGUACA GCUUGTT-3′; and Negative control: sense, 5′-GCGAC GAUCUGCCUAAGAUTT-3′ and antisense, 5′-AUCUUA GGCAGAUCGUCGCTT-3′. siRNA duplexes for *in vivo* silencing were 5′-cholesterol and 2′-O-Methyl oligonucleotide (2′-OMe) modified to increase stability.

### *In vitro* gene silencing

HepG2, SMMC-7721, or Huh7 cells were seeded into 6-well plates, 24-well plates or 96-well plates and cultured for 24 h. The cells were then incubated at 37°C for 6 h with an siRNA duplex mixed with RFect siRNA transfection reagent and lipofectamine (Bio-trans, Shanghai, China) in serum-free medium at a final siRNA concentration of 300 nM. After transfection, cells were cultured in complete culture medium for 72 h.

### RNA isolation and qPCR

Total RNA was extracted using RNAisoPlus (TaKaRa, Dalian, China) and transcribed into cDNA using a PrimeScript^™^ RT Reagent Kit (TaKaRa) according to the manufacturer's protocols. GP73 mRNA expression was quantified via quantitative real-time PCR (qRT-PCR) using a 7500 Real-Time PCR system (ThermoScientific, MA, USA). qPCR was performed using SYBR Premix Ex Taq^®^ (TaKaRa) with the following GP73 primers: 5′-CAGCGCTGATTTTGAGATGAC-3′ and 5′-ATGATCCGTGTCTGGAGGTC-3′. GP73 mRNA levels were normalized to β-actin with the following primers: 5′-TTCCAGCCTTCCTTCCTGGG-3′ and 5′-TTGCGCTCAGGAGGAGCAAT-3′. PCR parameters consisted of an initial incubation of 60 s at 95°C, followed by 35 cycles at 95°C for 20 s each and 1 cycle each at 95°C for 15 s, 60°C for 60 s and 95°C for 15 s.

### MTS proliferation assay

An MTS assay was used to assess proliferation, with cells seeded into 96-well plates at a concentration of 5 × 10^3^ cells/ml. After transfection, cells were cultured with 100 μl complete medium for 72 h. Next, 100 μl of MTS solution (Promega Biosciences, San Luis Obispo, CA, USA) mixed with DMEM complete medium (1:9 ratio) was added to each well and incubated for 4 h at 37°C. Results were quantified using a microplate reader (Molecular Devices) at an absorbance of 490 nm.

### Anchorage-independent cell colony formation assay

Anchorage-independent proliferation was examined in all three cell lines following GP73 silencing. For this assay, cells were seeded into 6-well plates (1 × 10^4^ cells/ml). Following transfection, cells were extracted using a 10% Basal Medium Eagle (BME) (Sigma-Aldrich Co., St. Louis, USA)/Bacto agar (Becton Dickinson, Bedford, USA) mixture at a 1:3 ratio as previously described [18] and a final volume of 1 ml with approximately 5 × 10^3^ cells. Plates were prepared by adding 3 ml of warm agar mix (43°C) to each well, with the 10% BME contained cells on the top agar after the bottom agar cooled. The mixture was then incubated at 37°C and 5% CO_2_ for 6 d and the colonies were counted at a 40 × magnification.

### Immunoblot analysis

Western blot analysis was performed to detect proteins within the MAPK signaling pathway and factors relating to cell cycle progression following GP73 silencing. Whole cell lysates were prepared in 1× RIPA lysis buffer (Millipore, CA, USA) containing a 1× protease and phosphatase inhibitor cocktail (Thermo Scientific, MA, USA). Protein concentrations were determined using a BCA protein assay kit (Thermo Scientific, IL, USA) and Nanodrop 2000 according to the manufacturer's protocols. Equal lysate volumes (varying from 20–80 μg) were separated via sodium dodecyl sulfate-polyacrylamide gel electrophoresis (SDS-PAGE), transferred onto a polyvinylidene difluoride (PVDF) membrane and blocked with 5% BSA in ddH_2_O for 1 h at room temperature. After blocking, PVDF membranes were incubated overnight with primary antibodies diluted in 1× TBST with 5% BSA at 4°C. Membranes were then washed three times with 1× TBST and incubated with horseradish peroxidase (HRP)-conjugated secondary antibodies (EarthOx, CA, USA) for 1 h at room temperature. Blots were then washed three times with TBST and visualized with an ECL-HRP chemiluminescence kit (Biological Industries, BI, Israel). Primary rabbit polyclonal antibodies were as follows: Anti-p44/42 MAPK(Erk1/2), anti-p-p44/42 MAPK(Erk1/2), anti-Akt, anti-p-Akt(Ser473), anti-Rb, anti-p-Rb(Ser780), anti-SAPK/JNK, anti-p-SAPK/JNK, anti-HSP27, anti-p-HSP27, anti-P38 MAPK, anti-p-P38 MAPK, anti-CDK2, anti-N-cadherin and anti-E-cadherin (Cell Signaling Technology, Beverly, MA) (1:2,000). Anti-β-actin rabbit polyclonal antibody was purchased from EarthOx, San Francisco, CA (1:3,000). Anti-GP73 rabbit polyclonal antibody was purchased from Prof. Aixia Zhang, Nanjing Medical University, Nanjing, China (1:2,000) [19].

### Immunofluorescence and confocal microscopy

Each well of a 24-well plate was seeded with 2 × 10^4^ cells and incubated for 24 h. Following siRNA transfection and a 72 h incubation, cells were fixed in 4% paraformaldehyde for 30 min followed by cellular permeation with 0.3% Triton X-100 for 20 min. Samples were blocked with 10% FBS in 1× PBS for 30 min and incubated overnight with primary antibodies at 4°C. Primary antibodies used here included rabbit polyclonal anti-N-Cadherin and anti-E-Cadherin antibodies (Abcam; 1:400) and mouse monoclonal anti-GP73 (Abnova; 1:100). Samples were then washed three times with 1× PBS and incubated for 1 h at 37°C with Alexa Flour^®^ conjugated secondary antibodies (Life Technologies, Grand Island, NY, USA) and DAPI (Sigma-Aldrich Co, St. Louis, MO). Cells were then washed three times with 1× PBS and samples were sealed under coverslips with transparent nail polish. Cells were examined using a confocal microscope (Carl Zeiss Jena, Oberkochen, Germany) and analyzed using Image-J.

### Cell cycle assessment via flow cytometry

Cell cycle related elements were assessed via flow cytometry following GP73 silencing. Briefly, cells were mixed with serum-free medium, seeded in 6-well plates (1 × 10^5^ cells/well) and incubated for 24 h to synchronize the cell cycles. After transfection, cells were cultured in complete medium for 72 h. Approximately 1 × 10^6^ cells were scattered into 1 ml of ethanol and fixed overnight. Cells were then stained using PI solution at 37°C for 30 min and analyzed via flow cytometry.

### Human hepatocellular xenograft tumor model

Male BALB/c nude mice (4.5 weeks old) were purchased from Slac Laboratories (Shanghai, China). All the animals received care in compliance with guidelines in the Guide for the Care and Use of Laboratory Animals. The procedures were approved by Zhejiang University Laboratory Animal Center. Xenograft tumor models were generated by subcutaneously injecting 100 μl of HepG2 cells (5 × 10^6^ cell/mouse) or SMMC-7721 cells (3 × 10^6^ cells/mouse) mixed in 50% Matrigel (Becton Dickinson, Bedford, MA) into the right fat pads of nude mice [15].

### *In vivo* gene silencing

When tumor volumes were about 50 mm^3^, the mice were randomly divided and treated with siGP73 1, siGP73 2, negative control (1 mg/kg) and normal saline (NS) every three days by tail vein injection. The perpendicular diameters of the tumors were measured using calipers before each injection. The estimated volume was calculated based on the following equation: tumor volume = ½ × width^2^ × length. The weights of nude mice were also measured before each injection as previously described [15, 16]. Two days after the last treatment, the animals were sacrificed and the tumors were excised for immunohistochemical analysis with GP73 detected. Serum was also collected for cytokine and interferon analysis [15].

### Immunohistochemistry

Tumor tissues derived from nude mice were fixed in formalin solution for more than 24 h. For IHC staining, tissues were sectioned (4 μm), mounted on positively charged glass slides (Thermo Scientific, MA, USA), baked, deparaffinized and rehydrated as previously described [14]. Antigen retrieval was completed by heating slides in citrate buffer (10 mmol/L; pH 6.0) for 20 min in a pressure cooker. Sections were incubated overnight at 4°C with GP73 rabbit polyclonal antibody (1:1,000) and then washed with PBS. Washed sections were incubated with HRP-conjugated goat anti-rabbit IgG secondary antibodies (EarthOx, CA, USA) at room temperature for 30 min and visualized with 3, 3′-diaminobenzidine (Vector Laboratories, Burlingame, CA, USA.

### Mouse cytokine and interferon analysis

Two days after the last treatment, serum was isolated and assayed for mouse IFN-γ, TNF-α and IL-6 using a quantitative enzyme-linked immunosorbent assay (ELISA) kit (Neobioscience, Beijing, China) according to the manufacturer's instructions. Samples were quantified using a Bio-Rad microplate reader (Hercules, CA, USA) at an absorbance of 450 nm [15].

### Transwell migration and invasion assays

6.5 mm Transwell^®^ with 8.0 μm Pore Polycarbonate Membrane Coated inserts were purchased from Corning (NY, USA). Cells were seeded in 6-well plates (2 × 10^5^ cells/well) and incubated for 24 h. After transfection, cells were cultured in complete medium for an additional 24 h. The cellular density was adjusted to 1 × 10^5^ cells/ml to account for non-adhered cells. For the invasion assay, 1 × 10^4^ cells in 100 μl serum-free DMEM were seeded in the upper chamber of the insert, with 15% Matrigel tiled on the membrane of the upper chamber; 800 μl DMEM containing 10% FBS was added to the lower chamber and incubated for 2 d. The medium and cells were then removed from the upper chamber using cotton swabs with 1× PBS. The cells were fixed with 800 μl methanol for 30 min, stained with a 0.5% crystal violet solution for 2 h, washed with 1 × PBS and counted under a microscope [20].

## References

[1] Kladney RD, Bulla GA, Guo L, Mason AL, Tollefson AE, Simon DJ, Koutoubi Z, Fimmel CJ (2002). GP73, a novel Golgi-localized protein upregulated by viral infection. Gene.

[2] Ba MC, Long H, Tang YQ, Cui SZ (2012). GP73 expression and its significance in the diagnosis of hepatocellular carcinoma: a review. International journal of clinical and experimental pathology.

[3] Kim HJ, Lv DD, Zhang Y, Peng T, Ma XJ (2012). Golgi phosphoprotein 2 in physiology and in diseases. Cell & Bioscience.

[4] Gong Y, Long Q, Xie H, Zhang T, Peng T (2012). Cloning and characterization of human Golgi phosphoprotein 2 gene (GOLPH2/GP73/GOLM1) promoter. Biochemical and biophysical research communications.

[5] Zhao J, Guo LY, Yang JM, Jia JW (2015). Sublingual vein parameters, AFP, AFP-L3, and GP73 in patients with hepatocellular carcinoma. Genetics and molecular research.

[6] Yorita K, Ohno A, Kataoka H (2014). Emerging histopathological prognostic biomarkers in hepatocellular carcinomas. Personalized Medicine Universe.

[7] Zhou Y, Yin X, Ying J, Zhang B (2012). Golgi protein 73 versus alpha-fetoprotein as a biomarker for hepatocellular carcinoma: a diagnostic meta-analysis. BMC cancer.

[8] Wei H, Zhang J, Li H, Ren H, Hao X, Huang Y (2014). GP73, a new marker for diagnosing HBV-ACLF in population with chronic HBV infections. Diagnostic microbiology and infectious disease.

[9] Hu L, Yao W, Wang F, Rong X, Peng T (2014). GP73 is upregulated by hepatitis C virus (HCV) infection and enhances HCV secretion. PloS one.

[10] Wei H, Hao X, Li B, Li X, Hou J, Qiao Y (2013). GP73 is a potential marker for evaluating AIDS progression and antiretroviral therapy efficacy. Molecular biology reports.

[11] Kladney RD, Tollefson AE, Wold WSM, Fimmel CJ (2002). Upregulation of the Golgi Protein GP73 by Adenovirus Infection Requires the E1A CtBP Interaction Domain. Virology.

[12] Zhang YL, Zhang YC, Han W, Li YM, Wang GN, Yuan S, Wei FX, Wang JF, Jiang JJ, Zhang YW (2014). Effect of GP73 silencing on proliferation and apoptosis in hepatocellular cancer. World journal of gastroenterology.

[13] Kawashima M, Kawakita T, Higa K, Satake Y, Omoto M, Tsubota K, Shimmura S, Shimazaki J (2010). Subepithelial corneal fibrosis partially due to epithelial-mesenchymal transition of ocular surface epithelium. Molecular vision.

[14] Liu Y, Zhu YH, Mao CQ, Dou S, Shen S, Tan ZB, Wang J (2014). Triple negative breast cancer therapy with CDK1 siRNA delivered by cationic lipid assisted PEG-PLA nanoparticles. Journal of controlled release.

[15] Yang XZ, Dou S, Sun TM, Mao CQ, Wang HX, Wang J (2011). Systemic delivery of siRNA with cationic lipid assisted PEG-PLA nanoparticles for cancer therapy. Journal of controlled release.

[16] Hou J, Lin L, Zhou WP, Wang ZX, Ding GS, Dong QZ, Qin LX, Wu XB, Zheng YY, Yang Y, Tian W, Zhang Q, Wang CM (2011). Identification of miRNomes in human liver and hepatocellular carcinoma reveals miR-199a/b-3p as therapeutic target for hepatocellular carcinoma. Cancer cell.

[17] Renáta V, Abul I, Michael B, Jalees R, Nuria L, Elizaveta B (2015). Increased mitochondrial function downstream from KDM5A histone demethylase rescues differentiation in pRB-deficient cells. Genes & Development.

[18] Borowicz S, Van Scoyk M, Avasarala S, Karuppusamy Rathinam MK, Tauler J, Bikkavilli RK, Winn RA (2014). The soft agar colony formation assay. Journal of visualized experiments.

[19] Zhang A, Cao B (2012). Generation and characterization of an anti-GP73 monoclonal antibody for immunoblotting and sandwich ELISA. Journal of biomedical research.

[20] Tsai CF, Hsieh TH, Lee JN, Hsu CY, Wang YC, Lai FJ, Kuo KK, Wu HL, Tsai EM, Kuo PL (2014). Benzyl butyl phthalate induces migration, invasion, and angiogenesis of Huh7 hepatocellular carcinoma cells through nongenomic AhR/G-protein signaling. BMC cancer.

